# Acceptability and Initial Adoption of the Heart Observation App for Infants With Congenital Heart Disease: Qualitative Study

**DOI:** 10.2196/45920

**Published:** 2023-04-05

**Authors:** Elin Hjorth-Johansen, Elin Børøsund, Ingeborg Martinsen Østen, Henrik Holmstrøm, Anne Moen

**Affiliations:** 1 Neonatal Intensive Care Unit Division of Children and Adolescent Medicine Oslo University Hospital Oslo Norway; 2 Institute of Clinical Medicine Faculty of Medicine University of Oslo Oslo Norway; 3 Department of Digital Health Research Division of Medicine Oslo University Hospital Oslo Norway; 4 Department of Nursing and Health Sciences Faculty of Health and Social Sciences University of South-Eastern Norway Drammen Norway; 5 Department of Cardiology Division of Children and Adolescent Medicine Oslo University Hospital Oslo Norway; 6 Department of Public Health Science Institute of Health and Society, Faculty of Medicine University of Oslo Oslo Norway

**Keywords:** congenital heart disease, readiness for discharge, mobile app, follow-up, health services, mHealth

## Abstract

**Background:**

Approximately 1% of all infants are born with a congenital heart disease (CHD). Internationally CHD remains a major cause of infant death, some of which occur unexpectedly after a gradual deterioration at home. Many parents find it difficult to recognize worsening of symptoms.

**Objective:**

This study aims to report the acceptability and initial adoption of a mobile app, the Heart Observation app (HOBS), aiming to support parents’ understanding and management of their child’s condition and to increase quality in follow-up from health care professionals in complex health care services in Norway.

**Methods:**

A total of 9 families were interviewed on discharge from the neonatal intensive care unit and after 1 month at home. The infant’s primary nurse, community nurse, and cardiologist were also interviewed regarding their experiences about collaboration with the family. The interviews were analyzed inductively with thematic content analysis.

**Results:**

The analysis generated 4 main themes related to acceptability and adoption: (1) Individualize Initial Support, (2) Developing Confidence and Coping, (3) Normalize When Appropriate, and (4) Implementation in a Complex Service Pathway. The receptivity of parents to learn and attend in the intervention differs according to their present situation. Health care professionals emphasized the importance of adapting the introduction and guidance to parents’ receptivity to ensure comprehension, self-efficacy, and thereby acceptance before discharge (Individualize Initial Support). Parents perceived that HOBS served them well and nurtured confidence by teaching them what to be aware of. Health care professionals reported most parents as confident and informed. This potential effect increased the possibility of adoption (Developing Confidence and Coping). Parents expressed that HOBS was not an “everyday app” and wanted to normalize everyday life when appropriate. Health care professionals suggested differentiating use according to severity and reducing assessments after recovery to adapt the burden of assessments when appropriate (Normalize When Appropriate). Health care professionals’ attitude to implement HOBS in their services was positive. They perceived HOBS as useful to systemize guidance, to enhance communication regarding an infant’s condition, and to increase understanding of heart defects in health care professionals with sparse experience (Implementation in a Complex Service Pathway).

**Conclusions:**

This feasibility study shows that both parents and health care professionals found HOBS as a positive addition to the health care system and follow-up. HOBS was accepted and potentially useful, but health care professionals should guide parents initially to ensure comprehension and adapt timing to parents’ receptivity. By doing so, parents may be confident to know what to look for regarding their child’s health and cope at home. Differentiating between various diagnoses and severity is important to support normalization when appropriate. Further controlled studies are needed to assess adoption, usefulness, and benefits in the health care system.

## Introduction

Congenital heart disease (CHDs) are a birth defect affecting approximately 1% of newborns [1]. Approximately 25% of these infants have a severe CHD, and in Norway approximately 125 infants are born with a severe CHD each year [2]. Internationally, CHD is still a major cause of infant death [3], and around 10% of Norwegian children with severe heart disease die during the first 2 years of life [4]. Recent research shows that 29% of these deaths occurred unexpectedly unrelated to surgery, of which 60% after a gradual deterioration at home [5].

Many parents express difficulties in recognizing deterioration, and in situations where symptoms are detected, it may be difficult to describe them or decide what to do [6]. Comprehensive interstage home-monitoring programs using digitally transmitted assessments to a follow-up team support parents of the most vulnerable infants with single ventricle [7,8]. In Norway today, the population of this subgroup of infants with CHD remains small, but recent data show that other infants with CHD are also in need of supportive initiatives [5]. In Britain, an expert group recommended to develop an early warning tool to all infants with a severe CHD, which should be standardized nationally to improve discharge preparation and follow-up [3].

**Figure 1:**
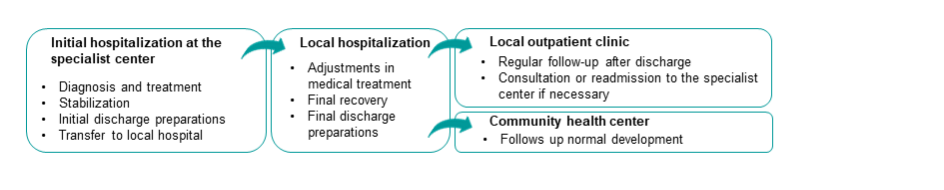
Health care services to infants with CHD initially treated at the specialist center in Norway. CHD: congenital heart disease.

Solutions adapted to mobile apps present novel opportunities to meet recommendations in follow-up for a more diverse group of infants with CHD. In recent years some initiatives have been started. In China, an app called “WeChat follow-up” supports parents with educational videos and information, telephone consultation, and chat with other parents using the app. This has shown to reduce worries and depression, improve quality of life, and increase knowledge about simple CHD [9,10]. An educational program including a mobile app with information to parents about infants with CHD was also developed in the United States. This app is informative but is not adapted to each child and not yet scientifically assessed [11]. Although these initiatives are promising, new strategies have to be compatible with the existing health services and personal, social, cultural, and organizational factors must be addressed [12-14]. In Norway, the Oslo University Hospital (OUH) is the only specialist center that performs surgery for children with CHD. In addition, they follow-up families in difficult cases and give advice and cooperate with local hospitals when needed (Figure 1). This gives the specialist center an opportunity to standardize an early warning tool and distribute it during their follow-up of 19 local hospitals. Hence, a project group at the specialist center developed the Heart Observation app (HOBS) in close collaboration with parents of infants with CHD and health professionals at local health care services [15].

HOBS is a complex intervention and The Medical Research Council recommends evaluating the feasibility of such interventions to ensure implementation [12]. A feasibility study should be designed to assess areas such as optimal content and delivery, acceptability, and adoption of the intervention among both recipients and deliverers of the intervention [12,14,16].

According to the Theoretical Framework of Acceptability, the concept of acceptability includes users’ attitude toward the intervention, burden of attendance, the extent to which the intervention fits with users’ value system, comprehension of the intervention, the effort to engage in the intervention, perceived effectiveness, and self-confidence to participate in the intervention [17]. Acceptability focuses on individual aspects, but when evaluating mobile health (mHealth) used by multidisciplinary teams in clinical care, it is important to have an additional focus on the interplay between technical, social, and organizational aspects. To do so, we have consulted a consolidated framework for adoption of mHealth [16]. Usability and optimization of HOBS content were reported previously [15]. The aim of this paper is therefore to present the feasibility study in which we report the results of assessment of acceptability and initial adoption among intervention deliverers and recipients to optimize implementation in an ongoing controlled trial.

## Methods

### The HOBS Intervention

#### Overview and Features

HOBS is developed as a capability-enhancing decision support tool and to support discharge preparations and follow-up at outpatient clinics [15].

HOBS has 5 main features: (1) My Child, (2) Information, (3) Contact, (4) Assessment, and (5) Summary (Figure 1).

#### My Child

**Figure 2:**
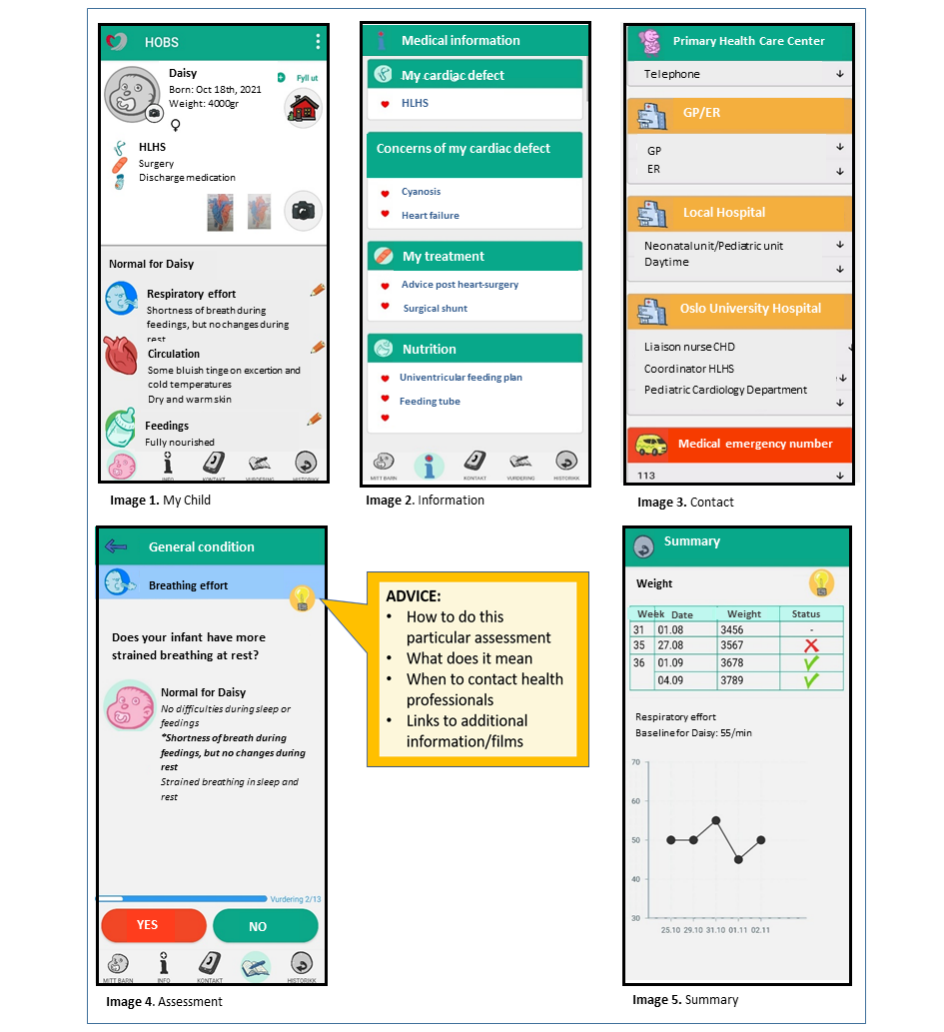
Features in The Heart Observation app (HOBS).

To personalize HOBS, the diagnosis, treatments, and need for monitoring and equipment are registered in “My Child” (image 1 in Figure 2). This provides parents with a personalized set of observations, information, and assessment questions. At discharge, parents do a final observation of their infant and store this information in the app as the normal baseline for the infant.

#### Information

This feature contains personalized information about the child’s diagnosis and its consequences, treatments, and use of equipment for monitoring, postoperative care, and nutrition (image 2 in Figure 2).

#### Contact

This feature contains explanations on where and when to contact health care professionals at the specialist center, locally and in emergency (image 3 in Figure 2). Parents add numbers to local services.

#### Assessment

This focuses on general condition, wound assessment, and weight gain. It presents personalized questions about changes from baseline regarding the child’s circulation, breathing, eating habits, and well-being (image 4 in Figure 2). An advice functionality guides parents on how to interpret signs.

#### Summary

This collects results from measurements and presents them in bar graphs and curves, and gives advice for the interpretation of weight gain (image 5 in Figure 2).

In this study, the HOBS intervention also included some support from health care professionals. These were (1) an overall introduction of features when uploading data to the app, (2) support to perform and choose the correct settings in “My Child” and to add observations in “Normal for my child” by bedside nurses, and (3) support to encourage parents to ask questions if they were uncertain about certain areas during hospital stay and before discharge.

### Study Design

HOBS is a complex intervention and therefore evaluated in several phases using a mixed methods design [12]. We have completed a usability study to adapt the app to the needs of parents and health care professionals [15]. This qualitative study aims to explore its feasibility, acceptability, and adoption to address further implementation in health services.

### Ethical Considerations

This study was approved by the Privacy Protection and Data security committee at Oslo University Hospital (project number 19/23041), and the Regional Committee for Medical and Health Research Ethics, South East, Norway (2019/1271). All parents signed an informed consent before taking part in the study and were informed that they had the opportunity to withdraw from the study at any time. Empirical findings from the study were presented as deidentified statements, according to Norwegian legislation.

### Inclusion and Introduction of HOBS to Parents

The eligibility criteria for participation among the parents were parenting an infant hospitalized with a severe CHD at the specialist center, gestational age above 34 weeks, having a smartphone, and able to read and write Norwegian. Bedside nurses invited families to learn about the project after the infant’s cardiac surgery or after the final diagnosis if no intervention was conducted before discharge. Those interested in participating received more information from the first author (EH-J). Parents agreeing to participate in the study signed a written consent form, before installing HOBS on their own phones. They received a 10–15-minute introduction of the main features of HOBS from the first author, and bedside nurses responsible for the patient helped parents with further personalization of the app to the infant. Because of COVID-19 precautions, mothers and fathers could only visit the unit 1 at the time, so most guidance to parents was given separately. As the severity of CHD varies among infants, and therefore the need for monitoring, each parent was advised to use HOBS (1) for assessments until they were confident in what to look for, (2) before consultations, and (3) if/when they felt unsure of the infant’s condition. They were encouraged to cooperate with nurses, cardiologists, and community nurses regarding their infant’s health using the app.

### Inclusion and Introduction of HOBS to Health Care Professionals

Nurses who were engaged in the guidance of parents and patient care at the specialist center received a 20-minute lecture about the purpose and use of HOBS, its features, and the tasks to complete together with the families. A test phone with the app was also available at the unit to make nurses confident with the features and content of HOBS. They were also encouraged to consult an e-learning course about the various features of HOBS and other resources available on the HOBS website, which was established to support health care professionals nationwide [18]. A checklist of nursing tasks to help parents prepare for discharge was available at the bedside. An electronic reminder in the hospital’s electronic system for monitoring and ordinations popped up 2 times a day for the nurses to ensure continuation of guidance before discharge. In local hospitals, nurses received a list of tasks to complete, but no lecture or test phone to practice. To support implementation of local follow-up, the first author called the local hospitals, providing information to the head nurse about the e-learning course on HOBS features, and asked for it to be shared among nurses in their hospitals. She also asked for identification of a nurse responsible for following up with the family that we interviewed after discharge from the local hospital.

On the day the infant was discharged from the specialist center, the first author contacted local cardiologists and community nurses following up with the family to share information about HOBS and asked for an opportunity to interview them 1 month after discharge. All health care professionals received the HOBS e-learning course in a link or as a Microsoft PowerPoint presentation, and were encouraged to include HOBS in their further cooperation with the parents.

### Data Collection

#### Interviews With Parents

Parents participated in 2 semistructured interviews via phone at the time of discharge from the specialist center and 1 month later (Table 2). The first author used a tape recorder and followed a semistructured interview protocol. The topics in the interviews during discharge were questions related to their experience with the introduction of and initial guidance with HOBS at the specialist center. For 5 families, the interviews were conducted when the child was still at a local hospital and for 4 while they were at home. The second interview focused on acceptability and adoption of HOBS during the follow-up of the child, and whether it affected their psychological adaption at home.

#### Individual Interviews and Focus Group Interviews With Health Professionals

Nurses and cardiologists at 6 local hospitals and community nurses at 8 different community centers were individually interviewed via phone by the first author (Table 1). She used semistructured interview protocols about their experience of cooperating with the family and views about implementing HOBS in their services.

In addition, we conducted 2 focus group interviews with nurses at the specialist center that followed the families in the study after the last family had completed the HOBS intervention. The moderator (EH-J) followed a semistructured interview guide and used a Microsoft PowerPoint presentation of HOBS features to refresh memories and avoid misconceptions. Experiences in using the app for guidance were discussed. One of the co-authors (IMØ) observed the interviews, noted ambiguity, and wrapped up the discussion to clarify the interpretations.

**Table 1:** Overview of interviews of parents and health care professionals.

Role/participation	Invited	Withdrew	Interviewed	Total minutes	Mean minutes	Range
Families first interview^a^	10	2^b,c^	8	96	12	7-14
Families second interview^d^	10	1^c^	9	162	18	10-26
Nurses	9	0	9	215	13	11-26
Cardiologists	9	2^c^	7	107	13	8-19
Community nurses	9	1^c^	8	122	16	9-22
Focus group	10	2^e^	8	165	N/A^f^	75-90

^a^8 mothers and 3 fathers.

^b^One family withdrew due to time constraints.

^c^Did not reply.

^d^9 mothers and 2 fathers.

^e^Time conflict.

^f^N/A: not applicable.

### Analysis

We used inductive thematic analysis as described by Braun and Clarke [19] for data analysis. EH-J and IMØ transcribed interviews and controlled the transcription consecutively during the study period. They wrote a summary of the 9 cases, consisting of interviews of parents and their health care professionals, to become familiar with the data (step 1). They interpreted, discussed ambiguity, and coded all interviews (step 2). EH-J organized interviews from parents and groups of health professionals separately into subthemes using NVivo (QSR International). IMØ generated themes from focus group interviews using Microsoft Word (step 3). After initial coding, both reviewed subthemes by condensing paragraphs from stakeholders’ experiences into meaningful units and restructured subthemes and themes (step 4). Finally, they merged themes from stakeholder groups to determine overarching themes (step 5). To clarify thoughts and inferences, they explained each theme and subtheme, and used quotes from participants to illustrate the subtheme of interest in a table. To validate interpretation, this final document was discussed with 2 other study authors (AM and EB) who were not involved in the initial development of HOBS. One mother of a child with CHD from the development group acknowledged the themes and interpretations as reasonable based on her own experience of HOBS and quotes from parents in this study. The Theoretical Framework of Acceptability and The Consolidated Framework for Adoption of mHealth supported further analysis of acceptability and adoption among parents and health care professionals to support further implementation of HOBS. The first author translated the quotes used in this article from Norwegian to English, and displayed them together in this paper to ensure agreement about translation and interpretation. We followed the “Consolidated criteria for reporting qualitative research” (COREQ) for writing this paper [20].

## Results

### Demographics and Clinical Characteristics of Parents and Infants

A total of 11 families were invited to participate from October 2020 to January 2021. All invited families agreed to participate; 1 family was not reachable after discharge and 1 infant had a quick recovery before discharge and thus were not eligible due to the scope of the study. For further details about the characteristics of parents and infants in the participating 9 families, see Table 2.

**Table 2:** Demographics and clinical characteristics of parents and infants (N=9).

Characteristics		Values
**Main caregiver in the first month**		
	Mother, n (%)	9 (100)
Main caregiver age, median (range)		31 (27-38)
Years of education after mandatory school, median (range)		7 (3-9)
Families with siblings, n (%)		6 (67)
Single parents, n (%)		1 (11)
**Infant diagnosis, n (%)**		
	Antenatal diagnosis	2 (22)
	Postnatal diagnosis	6 (67)
	Diagnosis after discharge from the maternity ward	1 (11)
**Infant treatment^a^, n (%)**		
	Surgery	6 (67)
	Catheterization	2 (22)
	Waiting for surgery	4 (44)
	Treatment with drugs after discharge	4 (44)
**Hospital stay, median (range)**		
	Total days of admission at the specialist center	12 (7-21)
	Age of infants at the introduction of HOBS^b^ (days)	8 (2-51)
	Days with HOBS before discharge from the specialist center	6 (2-9)
	Days at a local hospital before discharge	1 (0-7)
	Consultations with a liaison nurse	2 (0-3)
	Consultations with a psychologist	2 (0-3)
**Follow-up after discharge, median (range)**		
	Days of follow-up from the local hospital after discharge	0 (0-21)
	Consultations with a cardiologist after discharge	2 (2-4)
	Consultations with a community nurse	3 (2-4)
	Days from discharge to the second interview	38 (30-44)

^a^Infants could receive several treatments.

^b^HOBS: Heart Observation app.

### Results From Qualitative Interviews

**Figure 3:**
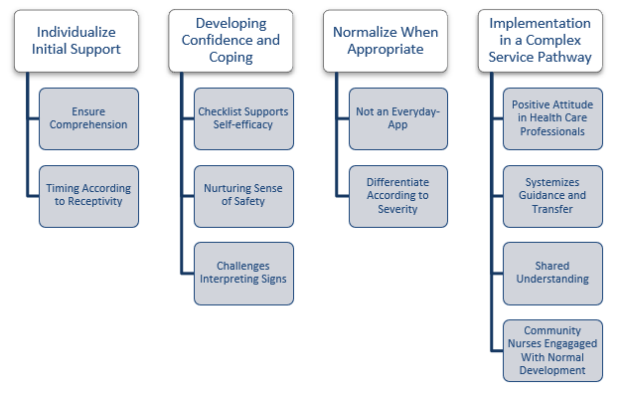
Themes and subthemes generated from parents' and health care professionals' experiences related to use of The Heart Observation app (HOBS).

The results represent analysis drawn from the data from families and their health care professionals. A total of 4 themes were identified: (1) Individualize Initial Support, (2) Developing Confidence and Coping, (3) Normalize When Appropriate, and (4) Implementation in a Complex Service Pathway. An overview of subthemes from parents and health professionals and their connection to themes are shown in Figure 3.

### Individualize Initial Support

#### Overview

In this study, infants had a broad spectrum of CHDs that led to various treatments, concerns, times of diagnosis, length of hospital stay, and days from birth to introduction of HOBS (Table 2). This variety demonstrated the heterogeneity of considerations, including specific concerns to be aware of, parents’ psychological state, initial receptivity of information, and interaction with health care professionals at the specialist center. Hence, individualizing the initial support was important to facilitate parents’ acceptability and experience of usefulness of HOBS. Topics regarding “Individualize Initial Support” were sorted into 2 subthemes: Ensure Comprehension and Timing According to Receptivity.

#### Ensure Comprehension

HOBS was seen as intuitive and easy to use by all parents, and most parents thought that the introduction and guidance to use the app were sufficient. Nevertheless, 2 parents of children with additional concerns, such as expected development of heart failure and cyanosis, mentioned a need for reassurance of comprehension and specific training in assessments. They asked for repetition of and focus on assessments before discharge:

...I think it was very nice that we went through it thoroughly first, and did a review with the nurses afterwards. (...) It would certainly have been nice to go through that (the assessments), maybe twice, when I was at the specialist center, to become even more confident about that part.

Mother 9

Based on experiences of parents’ comprehension, nurses suggested individualizing guidance to parental receptivity, providing parts of information and guidance over several occasions as well as a checkup before discharge to ensure comprehension. Preferably, experienced nurses who have both knowledge of concerns regarding CHD and pedagogical skills to adjust training to parents’ readiness should give the guidance. Although information is available in the app, they suggest ensuring parents have the correct settings in “My Child” and parents’ comprehension of assessment with health care professionals before discharge to optimize utilization and correct use.

...Before the parents leave the hospital, you may have to go through the app several times, and nurses should have assessed the child together with the parents, really.

Focus group 1, nurse 1

#### Timing According to Receptivity

Parents were introduced to HOBS after surgery or after treatment plan was clarified if no surgery was necessary before discharge. Most parents mentioned the time of introduction and initial guidance regarding HOBS as appropriate. One mother who unexpectedly gave birth to a child with a severe CHD was introduced to HOBS after 2 days. She was overwhelmed and declined to receive guidance regarding app settings and observations before discharge. She had installed HOBS and entered the settings in “My Child” and baseline without help at home. At the second interview, she said:

... Yes, I think it was a bit close to having a sick child. I think it was a bit overwhelming for me. (...) I think in a way you should probably take it (the training) when you get home. When you're kind of ready.

Mother 4

Nurses at the specialist center also experienced variance in stress and receptivity among parents and noted that it was important to consider this to avoid increased burden in an already stressful situation. They had experienced that inappropriate timing could affect parents self-efficacy and attitude to relate to guidance:

...It’s nice to go through the app when things have calmed down a bit. Because I notice a big difference in what the parents are able to deal with.

Focus group 1, nurse 3

### Developing Confidence and Coping

#### Overview

Parents appreciated the information in HOBS as relevant, comprehensive, and available when needed. Most parents expressed that they developed confidence and coping skills by gaining control over observations of wound healing, weight gain, whom to contact if necessary, and where to find information if unsure of something using HOBS. Knowing their infants’ normal baseline was important and gave confidence to detect changes. Nurses at local hospitals and cardiologists in follow-up described parents as informed, answering questions confidently at the outpatient clinic. This potential positive impact on confidence and coping seemed to enhance accept and adoption. Aspects regarding this theme were divided into 3 subthemes: Checklist Supports Self-efficacy, Nurturing Sense of Safety, and Challenges Interpreting Signs.

#### Checklist Supports Self-efficacy

HOBS intends to teach parents what to look for and helps them to decide whether they should contact their providers if they are uncertain about their infant’s condition. Most parents found that the checklist in HOBS helped them to assess the infant; they kept it as a guide in the back of their mind, and for some, it contributed to act on symptoms. A mother described how the HOBS assessment function supported her decision making in times of uncertainty:

...Because he cried a lot for a while, and I was not sure if it was normal baby needs or something with the heart. (...) I did the checklist inside the app and read that description about what to do. So it felt better, and I was not that worried.

Mother 1

Only 1 infant needed treatment for deterioration during the study period. On this occasion, HOBS supported decision making. The infant’s father said that he and his wife suspected a developing heart failure, and because their suspicion was supported/confirmed by features in HOBS, they contacted health professionals:

...It was actually, because they reduced his medication. So then, we saw clear symptoms of heart failure. (...) We did not have to wonder what it was.

Father 10

#### Nurturing Sense of Safety

To add focus on symptoms of deterioration in discharge preparation, instead of just telling parents to treat the infant as normal, may increase stress and anxiety, and thereby the burden of using the app. However, most parents indicated that HOBS increased their sense of safety when they were asked how using HOBS affected them. As one mother said:

...Absolutely no stress connected to the app at all. Very nice tool. And if there had been problems, or if he [the infant] had had any challenges in relation to an assessment, then it would have been used even more, I am absolutely sure of that.

Mother 3

Nevertheless, 1 mother mentioned that actually doing the assessment was a bit stressful, but knowing how to do it increased her self-confidence:

...There is a bit of stress in this, (...) But, I think it would possibly have been more stressful if I didn't know what to look for.

Mother 2

#### Challenges Interpreting Signs

Although 8 out of 9 parents coped well and felt confident about interpreting signs, the mother who did not receive guidance at the hospital expressed that the assessment of the infants’ crying and amount of vomiting turned normal changes into disease-related changes. The community nurse following this family also reflected on this as a challenge because most infants normally go through some weeks of increased restlessness after delivery. Hence, it could be difficult to relate to such symptoms. This perceived incoherence increased the burden of using HOBS to this mother and made her anxious:

...The assessment part seems to me to be challenging at times, because one question is whether the child cries more than usual. And hey, it's him, and I think there's something wrong with the heart right away.

Mother 4

### Normalize When Appropriate

#### Overview

After the initial use of HOBS at the local hospital, further use was influenced by parents’ aspiration to be a normal family. Parents as well as health care professionals focused on the importance to normalize daily living and individualize use of HOBS according to severity after discharge. This theme was divided into 2 subthemes: Not an Everyday App and Differentiate According to Severity.

#### Not an Everyday App

Many parents reflected on their initial anxiety to go home, of being alone with a newborn child with CHD following diagnosis, and were positive about using HOBS after the initial introduction to meet their needs. However, when the infant’s situation was stabilized and parents felt confident in what to look for, many chose to skip assessments due to time constraints or said they forgot to do it.

…It has been my biggest worry to go home when everyday life comes and I am all alone with him. Well, it is very much like that security whether I use it or not somehow, so know that I have it, I keep it in mind as an extra bit of security then.

Mother 6

Several parents expressed that they wanted to leave the illness behind when they left the hospitals, and return to normal everyday life after discharge. All parents expressed in some way that HOBS is not an “everyday app” and they had chosen to put HOBS away, and only use it if something came up that required necessary attention.

...It has not scared us. I actually feel quite safe. It is the app. The app is really quite brilliant when you need it, but when he [the infant] is stable and fine, we don't need it in the same way, but in times when it has been a bit uncertainty and we have something we wonder about, it has been very nice to have as a source of information.

Father 10

#### Differentiate According to Severity

Most cardiologists and community nurses emphasized the need to normalize the situation regarding stabilized infants. The cardiologists requested that the amount and type of assessments should be based on the infants need and on how severe the cardiac disease was:

...So in a way, for those infants who are developing heart failure or have an oxygen saturation of 75%, it is more relevant perhaps, but this baby is doing so very well.

Cardiologist 3

### Implementation in a Complex Service Pathway

#### Overview

Parents and their infants must relate to several health care providers in a complex pathway through different services. Time to learn to use the app varied, and after discharge, parents had 2-4 consultations with the outpatient clinic and 2-4 consultations at the community health care center during the study period of 1 month (Table 2). Topics regarding implementation in the service pathway were sorted into 4 subthemes: Positive Attitude in Health Care Professionals, Systemizing Guidance and Transfer, Shared Understanding, and Community Nurses Engaged With Normal Development.

#### Positive Attitude in Health Care Professionals

Nurses at the specialist center and locally were enthusiastic toward the content and focus of the app. They thought HOBS would reassure parents and be helpful in their own work. Health care professionals outside the specialist center only had information about HOBS through e-learning and Microsoft PowerPoint presentations, and they asked for access to the actual app prior to further implementation. Parents mentioned that they had to show local health care professionals how they used HOBS and most parents found it positive to share and had a positive attitude about the app:

...And well, they do not know the app very well, but they are very positive when they have used it a bit and I have just let them have my phone and check it out.

Mother 6

#### Systemizing Guidance and Transfer

Many cardiologists mentioned that they had time constraints during consultations and appreciated that competent nurses introduced HOBS to parents. Two of the cardiologists saw a potential that HOBS could enhance cooperation between services. Nurses, both at the specialist center and locally, shared that using HOBS together with parents in discharge preparation systemized guidance and helped them to know what to include in their discharge preparations. Hence, they thought it would improve the quality of discharge preparation.

...I guess I have guided them in a way in the past, but now I get a tool that I can use systematically which means that I do not leave anything out.

Nurse 7

In addition, nurses and community nurses pointed out that HOBS gave knowledge and opportunities to understand the complexity of CHD in an individual child, and the infants’ follow-up, if they knew the settings in HOBS for a particular infant.

...Because I am not that familiar with these heart children, and because there are different diagnoses, and different symptoms and different prospects for the future, I think it was very clear to see; -oh yes you have done that, then we can expect this.

Nurse 6

#### Shared Understanding

After discharge, cardiologists at the outpatient clinic focused on hemodynamics through echocardiographic ultrasound. In addition, it was important for them to receive information about how parents perceived the infant’s general condition. Most cardiologists noted that HOBS could contribute positively to the conversation about the infant’s condition:

...It is important how they (parents) perceive their child. (...) So, in that sense, this (HOBS) can help me with the assessment through the conversation with the parents.

Cardiologist 6

Parents also emphasized such shared understanding and a positive contribution to conversation:

...I have read the fine articles that were in the app, and I think they were very explanatory and very easy to understand for someone who is not a doctor. (...) Which means, that I understand the medical language a little better.”

Mother 6

Cardiologists emphasized that HOBS provides more specific observations, and that several parents using HOBS gave relevant answers to questions that concerned the cardiologist. One of the cardiologists mentioned that such joint attention could improve their dialog:

...It is good that they can use it to assess, so they have more objective assessments to give to us, and not just a feeling that things are going poorly.

Cardiologist 2

#### Community Nurses Engaged With Normal Development

Most community nurses have limited clinical experience in caring for infants with CHD and appreciated the individualized information they could receive using HOBS. They anticipated that vulnerable infants, postsurgical infants, or infants waiting for surgery might benefit from HOBS. At the same time, they expressed that the community health services should follow-up normal development not the cardiac disease.

...At the health center, we must have follow-up on the healthy part of the child to what is normal development and growth. So, I think this will be, in a way, between parents and the specialist health service and possibly a general practitioner.

Community nurse 9

This view reflects community nurses seeking to limit responsibility and support, and in some community nurses, it reflects low self-efficacy regarding interpretation of assessment. As one community nurse puts it:

...It is a bit difficult for us as community nurses. When to normalize and when to say, yes, this could be the heart defect.

Community nurse 4

## Discussion

### Principal Findings

The major finding of this feasibility study was that both parents and health care professionals regarded the content and functionality of HOBS as a positive addition to the health care system and follow-up. HOBS was considered feasible, acceptable, and potentially useful, especially when guidance was timed to individual needs and comprehension was ensured. Parents may then become confident, knowing what to look for, and be vigilant at home. Differentiated use according to the child’s condition supports appropriate normalization in less severe cases.

### Individualize Initial Support

Despite different viewpoints, parents and nurses shared the understanding that appropriate timing and guidance in individualized sessions were important. To give birth to a child with cardiac disease causes stress and anxiety in parents [21]. Discharge preparations are often challenging and may suffer from reduced ability to handle a new situation because of overwhelming feelings after delivery and during hospitalization [22-25]. Hence, an unfortunate timing and lack of training may reduce comprehension and utilization and eventually confidence to perform assessments in HOBS. Adapting the introduction and training to parents’ perceptivity to ensure comprehension and reduce the burden of attendance might optimize utilization in follow-up [17]. However, overdoing reminders and guidance in this situation may be unfortunate and reduce acceptability [14]. In general, visits to the outpatient clinic are frequent the first month, which makes it possible to ensure comprehension after discharge if necessary. In addition, health care professionals that offer coaching and use data presented by parents may enhance the adoption of services such as HOBS [14].

### Developing Confidence and Coping

Parents in this study considered the information content of HOBS relevant, comprehensive, and easily available. Such information serves as health education and if complemented with assessment functionality, it facilitates the adoption of mHealth in many studies, especially if personalized and received after initial diagnoses, such as HOBS [14]. Caring for a recently discharged infant with CHD requires an understanding and awareness about what to look for [3]. To be constantly aware and assess signs of deterioration might however be stressful, irrespective of apps used [26]. In this study, parents appreciated the sense of safety that the checklist of assessments provided. Hence, the intention to support confidence and coping seems to be achieved. At the same time, a single episode of deterioration in this study cannot verify whether HOBS will be effective to detect deterioration. However, when parents are confident in what to look for may give them an opportunity to normalize daily living and at the same time feel relaxed and secure because they have easy access to available and relevant information and a checklist to confer with [27]. The opposite may occur if parents are overwhelmed and this results in deficient introduction and guidance and hinders comprehension and self-efficacy [23].

### Normalize When Appropriate

A finding in this study was that parents said they reduced the number of assessments performed very soon, and user log reported in the previous usability study confirmed that parents had a median of 2 assessments at home during the first month [15]. Frequent consultations with health care professionals have reduced adoption in other studies [14]. Therefore, few assessments in this study may be explained by frequent consultations with cardiologists and community nurses during the study period. At the same time, parents expressed a wish to end assessments and focus on normal daily living when confident. This corresponds with studies of an early warning tool for parents of infants with CHD [27]. Nevertheless, an educational mHealth intervention for parents of a diverse group of infants with CHD showed that biweekly monitoring of vital signs over time did not reduce stress, anxiety, and adverse events [26]. To be constantly reminded of disease and symptoms may maintain anxiety [14]. Hence, a reduction in routine assessments might reduce the burden. Yet, well-educated and informed parents may cope well under such pressure [28]. Consequently, discontinued use of HOBS may not correlate with acceptability, considering that parents perceived HOBS as effective and intended to do assessments if necessary [29,30]. Our results indicate that parents were able to find the balance between awareness of symptoms and coping with their new situation. This supports the feasibility of HOBS as a tool for discharge preparation and decision support in times of uncertainty.

### Implementation in a Complex Service Pathway

One of the principal findings of this study was that health care professionals viewed parents who used HOBS as well informed and confident in assessing their child. Despite the knowledge about possible deterioration, most parents were able to normalize family life after discharge. HOBS was developed as an early warning tool to support parents to detect deterioration based on recommendations from an expert group [3]. A well-known concern among health care professionals is that parents may become more anxious if they have to assess and be aware of symptoms of worsening instead of treating their child as normal [31]. Such contextual factors might influence adoption and acceptability by health care professionals and thereby effectiveness of the intervention [12]. In this study, health care professionals emphasized a possibility to differentiate use according to the condition of the child. The flexibility to adapt the assessments to the parents’ total burden may facilitate acceptability by making the intervention compatible with their own ethical view [17]. This may promote successful integration into existing services and thereby the adoption among health care professionals [13]. For parents of the most vulnerable infants, awareness of symptoms might not be enough and the infant’s cardiologist should recommend extending assessments over time [32]. Whether parents maintain the ability to detect deterioration even if they do not use the app regularly is currently uncertain. The adoption, use, and effects of HOBS among both parents and health care professionals need further evaluation over time.

As recommended for patient-centered approaches to mHealth adoption, we initially focused on integrating the intervention into the patient families’ journey all through health services [14]. This integration may explain acceptance and adoption reported in this study. In addition, our results show that different health care professionals have different and additive experiences and views of how the app may contribute.

Nurses were most enthusiastic and wanted to use the app to systemize and ensure quality in their guidance of parents. Such an expectation of improved parent education may encourage their adoption and further adherence to HOBS. However, nurses in the focus groups were concerned with general nurses’ ability to guide parents as intended. They suggested establishing an expert group to give the guidance, which is in line with recommendations to enhance adoption [13,14].

mHealth solutions such as HOBS are more likely to be adopted by clinicians if it empowers their patients [13]. In this study, cardiologists had little experience using HOBS but agreed to including HOBS into their services. This was supported by the experience of confident parents and enhanced communication regarding their infant’s general condition. At the same time, they also emphasized differentiation according to the child’s condition, which might reflect a conditional acceptance based on expected usefulness and management by parents in different cases.

However, community health nurses were more hesitant to adopt HOBS as a tool in daily consultations. One explanation may be that condition-specific solutions do not fit with their existing workflow and responsibility. Another explanation may be a lack of competence in CHD with fear of exposing knowledge gaps [13]. At the same time, they expressed positive attitude toward HOBS and were eager to learn and use it to increase their competency.

The positive attitude and perceived effectiveness of the app among health care professionals are beneficial regarding future implementation and adoption of the intervention [13,17]. In the ongoing controlled trial, adoption of the HOBS intervention will be assessed among both parents and health care professionals [33].

### Implications for Practice

**Figure 4:**
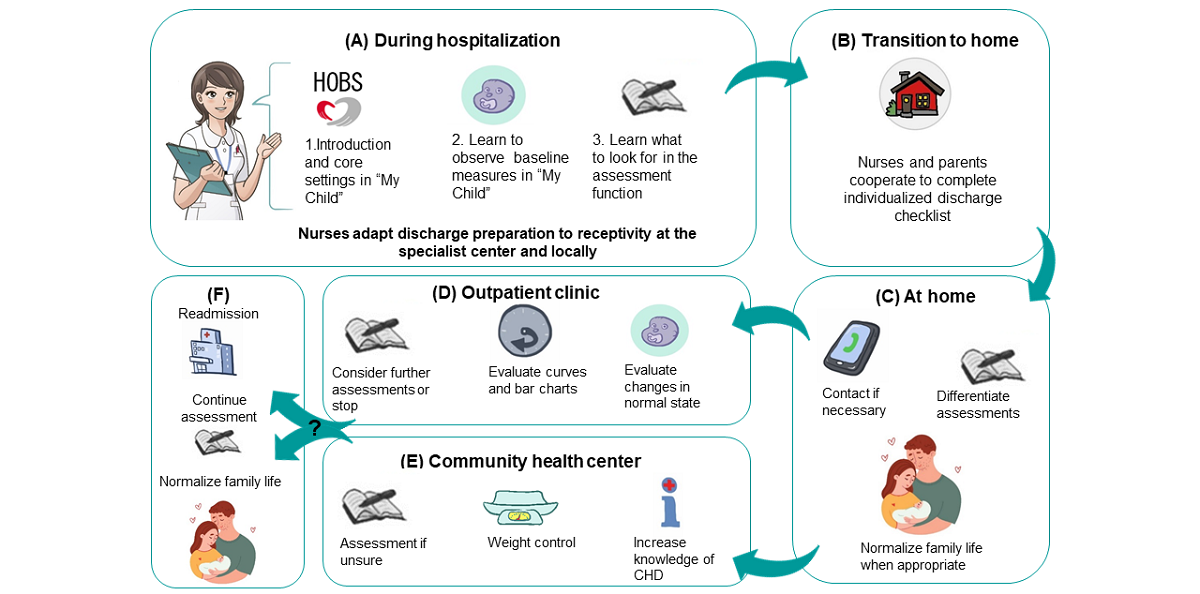
Implementation strategy for The Heart Observation app intervention in the upcoming controlled trial based on this feasibility study. Source of illustrations: Shutterstock and FluHartberg.

This feasibility study has explored acceptability and initial adoption of an app (HOBS) to identify factors that might influence its implementation in health care services. Based on these experiences, we have adjusted the strategy for implementation. As shown in Figure 4, parents are introduced on time to HOBS through 3 main areas during hospitalization (Figure 4A): (1) Introduction and core settings, (2) Observations in “Normal for my child,” and (3) Assessments of deterioration. Next, completion of discharge formalities is performed with the embedded checklist (Figure 4B). After discharge parents perform assessments according to severity (Figure 4C) and collaborate with the outpatient clinic regarding comprehension and results, and consider further assessments (Figure 4D). The community health center receives HOBS as a knowledge base and supports parents in assessments if they are unsure of their infant’s condition (Figure 4E). Further use is adapted according to the infant’s condition (Figure 4F). To support health care professionals, we offer a health care version of HOBS and an e-learning course with advice on how to guide parents. In addition, information in the patient journal about settings and guiding tasks to fulfill is sent from the specialist center to local hospitals. All these parts of the intervention program aim to ensure readiness for discharge; to improve parents’ confidence and coping; and to optimize comprehension, usefulness, and decision making. An ongoing controlled trial of the presented HOBS intervention assesses parents’ readiness for discharge, psychological adaptation, health literacy, and contact with health care services and compares them with standard care.

### Limitations

In this study, several limitations have to be addressed. First, our results of acceptability and adoption are promising but not conclusive. HOBS is a complex intervention with many components, and the context differs between health care centers, and parents may have different ability to use the app. This challenges any evaluation because the path to success might vary [12,34]. Our study sought to capture a broad spectrum of CHD diagnoses, concerns, and levels of health care. This resulted in interviews of local health care professionals with limited experience using HOBS. The results may therefore reflect anticipated rather than experienced effects. However, infants presenting with CHD are in general rare at most local hospitals and the unfamiliarity may reflect the situation in clinical practice. Second, the first author was deeply involved as content expert in the development of HOBS and in charge of the main part of data collection in the interviews, transcription, and analysis, which could increase the risk of researcher bias in the qualitative analysis. To reduce any potential bias, the coauthors contributed actively in the analysis. Third, it is possible that awareness about an upcoming interview may have affected motivation to use the app and it has been difficult to address negative experiences because the perceived providers (OUH) of the app are responsible for their infant’s further treatment. Fourth, we have followed parents and their health care professionals for a short period to evaluate acceptability and adoption and to address further implementation strategies.

### Conclusions

In general, parents and health care professionals felt HOBS as a feasible and positive addition to the health care system and follow-up. Our study shows that HOBS is accepted and useful when health care professionals guide parents and adapt the introduction and training to parents’ receptivity. Parents may then become more confident and know what to look for when caring for their infant with CHD at home. It will be important to differentiate use according to the child’s condition, and to support normalization through follow-up. Accounting for personal, social, and organizational factors will support feasibility and adoption of HOBS and its benefits. Further studies are needed to assess benefits and adoption in parents and health care professionals.

## Abbreviations

CHD: congenital heart disease
COREQ: Consolidated criteria for reporting qualitative research
HOBS: Heart Observation app
mHealth: mobile health
OUH: Oslo University Hospital

## Data Availability

The data sets generated during or analyzed during this study are not publicly available due to the Norwegian legislation and concerns of participants’ confidentiality.
