# Correction: Acceptability and Initial Adoption of the Heart Observation App for Infants With Congenital Heart Disease: Qualitative Study

**DOI:** 10.2196/70280

**Published:** 2025-01-10

**Authors:** Elin Hjorth Johansen, Elin Børøsund, Ingeborg Martinsen Østen, Henrik Holmstrøm, Anne Moen

**Affiliations:** 1 Neonatal Intensive Care Unit Division of Children and Adolescent Medicine Oslo University Hospital Oslo Norway; 2 Institute of Clinical Medicine Faculty of Medicine University of Oslo Oslo Norway; 3 Department of Digital Health Research Division of Medicine Oslo University Hospital Oslo Norway; 4 Department of Nursing and Health Sciences Faculty of Health and Social Sciences University of South-Eastern Norway, Drammen Norway; 5 Department of Cardiology Division of Children and Adolescent Medicine Oslo University Hospital Oslo Norway; 6 Department of Public Health Science, Institute of Health and Society Faculty of Medicine University of Oslo Oslo Norway

In “Acceptability and Initial Adoption of the Heart Observation App for Infants with Congenital Heart Disease: Qualitative Study” (JMIR Form Res 2023; 7:e45920) the authors noted that author Elin Hjorth Johansen's affiliations need to be adjusted.

The following affiliation was added for author Elin Hjorth Johansen:

^2^Institute of Clinical Medicine, Faculty of Medicine, University of Oslo, Oslo, Norway

The correction will appear in the online version of the paper on the JMIR Publications website on January 10, 2025, together with the publication of this correction notice. Because this was made after submission to PubMed, PubMed Central, and other full-text repositories, the corrected article has also been resubmitted to those repositories.

